# Development and Validation of an Unethical Professional Behavior Tendencies Scale for Student Teachers

**DOI:** 10.3389/fpsyg.2021.770681

**Published:** 2021-12-02

**Authors:** Jing Wang, Xin-qiang Wang, Jia-yuan Li, Cui-rong Zhao, Ming-fan Liu, Bao-juan Ye

**Affiliations:** ^1^School of Psychology, Center for Mental Health Education and Research, Institute of Psychological Technology Application, Jiangxi Normal University, Nanchang, China; ^2^Mental Health Education for College Students, Wuchang Shouyi University, Wuhan, China; ^3^School of International Education, Sichuan International Studies University, Chongqing, China

**Keywords:** student teachers, unethical professional behaviors, measurement invariance, gender differences, scale development

## Abstract

Teacher’s unethical professional behaviors affect students’ physical and mental health. Prevention should start with student teachers, but empirical research is lacking in China. This study surveyed over 2,000 student teachers from China to examine the psychometric properties of a student teachers’ unethical professional behavior tendencies scale which revised by primary and secondary school teachers’ unethical professional behavior tendencies scale. Exploratory and confirmatory factor analysis confirmed that a bi-factor model fit the data best. The final student teachers’ unethical professional behavior tendencies scale comprised four subscales, including a general factor (unethical professional behaviors) and four special factors (perfunctory attitude and carelessness, insults and discrimination, unfairness, and using power for personal gain). The student teachers’ unethical professional behavior tendencies scale correlated negatively with their professional ethical values and positively with perceived frequency of unethical professional behaviors of college teachers around them. The data supported the scale’s measurement invariance across gender, and male student teachers scored significantly higher on unethical professional behavior tendencies than female student teachers. The findings suggest that the student teachers’ unethical professional behavior tendencies scale is a useful instrument for assessing student teachers’ unethical professional behaviors in China.

## Introduction

In recent years, frequent news reports of unethical professional behaviors by teachers have attracted considerable attention and discussion in society. Teachers’ unethical professional behaviors not only affect students’ physical and mental health but also influence the development of students’ careers and values (Arslan and Dinç, 2017; Zheng et al., 2017; Liu, 2021). Teachers’ unethical professional behaviors are developed while they are student teachers, and thus, cultivation of and attention to teachers’ morality needs to start before they begin the work (Eren and Rakıcıoğlu-Söylemez, 2017). Student teachers refer to a university student oriented to the teaching profession, trained in teaching skills, and involved in a school-based field experience (Zhang, 2014; Zhu et al., 2019). Student teachers belong to preservice teachers, and they are in the process of learning how to become formal teachers and lacking a lot of teaching experience, but they already have the tendency of teacher’s moral behaviors at this stage which behavioral patterns may predict their future behaviors (Barrett et al., 2012). Student teachers’ morality has a great influence on their future educational practice (Atjonen, 2012; Bosica et al., 2021).

Although existing courses about professional ethics could help instill high ideals in student teachers, negative aspects concerning unethical professional behaviors have been ignored (Cai, 2019; Zhao, 2019; Liu, 2021). To reduce unethical professional behavior tendencies of student teachers at the beginning of their training, it is necessary to develop a measure of student teachers’ unethical professional behavior tendencies. However, there are few studies on student teachers’ unethical professional behavior tendencies, and more studies mainly focus on primary and secondary school teachers who are already working (Ge, 2008; Barrett et al., 2012; Guo, 2017; Cai, 2019). Unethical professional behavior is a social behavior, its hierarchy is developed in the most frequently occurring sett, and it has a close relationship with the social culture (Whiting, 1980). Studies have shown that in the context of collectivist culture and interdependent culture, people respect authority and are required to obey the expectations of authority and the sociocultural environment (Kathrine, 2015; Malmi et al., 2020). Therefore, the present study sought to develop and validate an unethical professional behavior tendencies scale for Chinese student teachers, so as to help researchers enrich the assessment tools for student teachers’ unethical professional behavior tendencies and understand the characteristics of student teachers’ unethical professional behavior tendencies in the context of collectivist culture and interdependent culture.

### Definitions of Student Teachers’ Unethical Professional Behavior Tendencies

Unethical professional behaviors refer to behaviors in which practitioners violate professional ethics. Researches about teachers’ unethical professional behavior have always focused on two aspects. In terms of teaching, teachers’ unethical professional behavior has been considered as the behaviors which did not meet the teaching needs (Zhao, 2019; Eren and Rakcolu-Sylemez, 2021). In terms of life outside the school, teachers’ unethical professional behavior discussion has mainly focused on using power for personal gain (Eren and Rakıcıoğlu-Söylemez, 2017; Zhang, 2017). Although student teachers have limited work experience, students are the main people they provide services in fieldwork and their future careers. Students and teachers can communicate and interact both during teaching and in terms of life outside school settings. Therefore, student teachers’ unethical professional behavior tendencies refer to behavioral tendencies that are contrary to teachers’ professional ethics concerning student-teacher relations during teaching and life.

### The Dimensions About Student Teachers’ Unethical Professional Behavior Tendencies

Most research on teachers’ unethical professional behaviors has concerned in-service teachers in China (Ge, 2008; Guo, 2017; Zhang, 2017; Cai, 2019). Student teachers have a clear career orientation, and their unethical professional behavior tendencies involve students, as with in-service teachers (Eren and Rakıcıoğlu-Söylemez, 2017; Eren and Rakcolu-Sylemez, 2021). A series of empirical studies on the unethical professional behavior tendencies of in-service teachers and student teachers were summarized as follows: in terms of teaching, discussion of unethical professional behavior tendencies has mainly focused on perfunctory attitude, carelessness, and unfairness (Ge, 2008; Jiang, 2013; Guo, 2017; Zhang, 2017). Barrett et al.’s (2012) study in which participants are American teachers found that the unethical professional behavior tendencies of carelessness and unfair treatment of students are also widespread. Teachers’ seriousness and partiality in teaching are directly related to students’ academic achievement, and educational fairness is related to the long-term development of education (Allen et al., 2013; Brunila and Kallioniemi, 2018). In terms of life outside the school, a common unethical professional behavior involves that teachers using their power for personal gain (Zhao, 2009; Zhang, 2017). Such behavior is not only found in China but also provoked a public revolution in Turkey (Eren and Rakıcıoğlu-Söylemez, 2017). Because juveniles have immature values and are susceptible to influence, when teachers use their power to seek personal gains, it will affect students’ development and future achievements (Sirait, 2016; Liu, 2021). While occurring during teaching and in terms of life outside the school, teachers’ physical and mental insults and discrimination are a form of unethical professional behaviors with a significant impact on students. Several studies found that insults and discrimination toward students are some of the highest frequency unethical professional behaviors of primary and secondary school teachers, and this behavior not only directly harms students’ physical and mental health but also causes other students to become prejudiced against the victims (Ge, 2008; Zhang, 2017; Ssenyonga et al., 2019).

The unethical professional behaviors scale for primary and secondary school developed by Wang et al. (2019) include perfunctory attitude and carelessness (student teachers do not take education seriously during teaching), insults and discrimination (verbal humiliation or corporal punishment of students during study or life), unfairness (unfairly reward or punish students in their studies), and using power for personal gain (selling goods to the students or to be a tutor for gaining personal benefit). The scale is comprehensive and developed based on Chinese teachers. Therefore, this study will revise the scale to make it suitable for student teachers and use the scale to explore the psychometric properties of student teachers’ unethical professional behavior tendencies.

**FIGURE 1:**
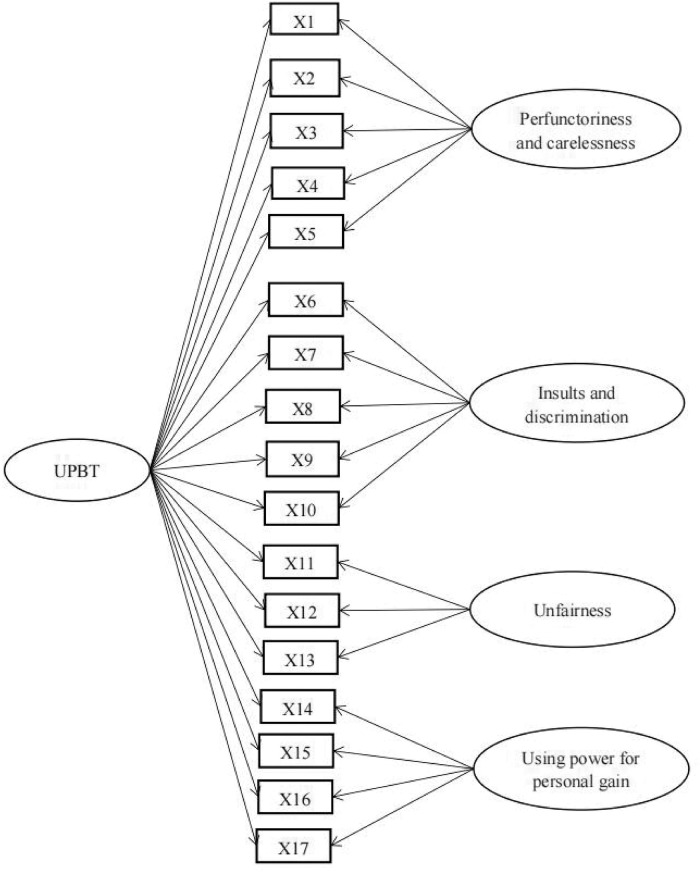
Hypothetical bi-factor model. UPBT = unethical professional behavior tendencies, 17 items were proposed in the procedure section and the item details were presented in Table 1.

Nearly all studies about teachers’ unethical professional behaviors applied factor analysis to the measurement tools used, and the subscales have been compiled according to the theoretical conception of unethical professional behaviors (Barrett et al., 2012; Eren and Rakıcıoğlu-Söylemez, 2017; Guo, 2017). This method ignores the commonalities and differences between the various dimensions. The widely used “bi-factor model,” also called the “general–special factor model,” assumes that there are both general and special factors. The general factor explains the common variance of all items, and the special factors interpret the common variance of subsets of items included in the general factor (Chen et al., 2006; Heubeck and Wilkinson, 2019). Bi-factor models are also particularly amenable to the estimation of model-based reliabilities for both global composite scores and subscale index scores (Gignac and Watkins, 2013). The four dimensions included in the unethical professional behaviors scale developed by Wang et al. (2019) have been found to be both independent and related to each other in previous studies (Barrett et al., 2012; Guo, 2017; Zhang, 2017), which accords with the assumptions of the bi-factor model. Therefore, this study proposed Hypothesis 1 that student teachers’ unethical professional behavior tendencies will fit a bi-factor model that includes a general factor (overall unethical professional behavior tendencies) and several special factors (refer to in Figure 1).

**TABLE 1:** The initial 17-item unethical professional behavior tendencies scale.

**Item**
^*^1. In some cases, treat students carelessly
(在某些情况下,敷衍塞责学生)
^*^2. In some cases, treat students’ homework carelessly
(在某些情况下,不认真批改作业)
^*^3. In some cases, teach courses carelessly
(在某些情况下,不认真备课或上课)
4. In some cases, lack of respect for students
(在某些情况下,不尊重学生)
5. In some cases, don’t safeguard the right and interests of students.
(在某些情况下,不保护学生的权利和利益)
^*^6. In some cases, ridiculing students
(在某些情况下,讽刺挖苦学生)
^*^7. In some cases, humiliating students who disobey school rules
(在某些情况下,在全班面前羞辱违纪学生)
8. In some cases, discriminating against students based on academic record
(在某些情况下,歧视学习成绩差的学生)
^*^9. In some cases, discriminating against students based on family conditions
(在某些情况下,歧视家庭条件差的学生)
10. In some cases, rasped insult to students
(在某些情况下,辱骂学生)
^*^11. In some cases, use violent disciplinary methods with students who disobey school rules
(在某些情况下,体罚或者变相体罚不听话的学生)
^*^12. In some cases, reward students unfairly
(在某些情况下,不公平地奖励自己喜欢的学生)
^*^13. In some cases, punish students unfairly
(在某些情况下,不公平地惩罚自己讨厌的学生)
^*^14. In some cases, run tutorial groups for money (在某些情况下,进行有偿家教)
^*^15. In some cases, use teachers’ power for personal gain
(在某些情况下,利用职务之便谋取自己的利益)
16. In some cases, evaluating students only from grades (在某些情况下,仅凭学生去评估学生)
^*^17. In some cases, selling school goods to students and their parents for money
(在某些情况下,向学生,家长推销学习用品以获取个人利益)

Items with * are the reserved items, others have been deleted; Items in brackets are the Chinese version.

### Professional Ethical Values and Unethical Professional Behavior Tendencies

Cognition affects human behavior, and ethical values are central to the human mind and crucial in determining human behavior (Fumagalli et al., 2010). Professional ethical behavior is closely related to practitioners’ ethical values. Their behaviors are constrained by regulations and law when they face ethical issues related to law, while what determines their decision-making in the face of legally unrelated ethical issues is the ethical values of practitioners (Cameron and O’Leary, 2015). Lee (2020) found that corporate ethical values are positively related to the ethical professional behaviors of individuals in work. Teachers who believe that violence can effectively regulate students’ behavior and improve their academic performance are more inclined to use violent discipline (Sirait, 2016). Ethical values have a far-reaching effect on student teachers’ unethical professional behavior tendencies. Cultivation of and attention to student teachers’ ethical values before they begin the work could prevent them from doing unethical professional behaviors in the future (Cai, 2019). Professional ethical values of student teachers also influence their unethical professional behavior tendencies, which are more correct professional ethical values, less unethical professional behavior tendencies (Barrett et al., 2012). The professional ethical value courses in universities are used for reducing student teachers’ unethical professional behavior tendencies in the future (Mathur and Corley, 2014). In addition, neuro-psychological studies on cognitive developmental changes indicate that the “executive function” of professional behavior is still improving in late adolescence and early adulthood (Yang et al., 2014). Student teachers are in the development process, and their professional ethical values and professional ethical behaviors are in the process of development. Student teachers’ unethical professional behavior tendencies are also influenced by their ethical values. It is necessary to research the relationship between the professional ethical values and professional behavior tendencies of student teachers, and this study proposed Hypothesis 2 that student teachers’ ethical values are negatively correlated with their own unethical professional behavior tendencies.

### Perceived Frequency of Unethical Professional Behaviors and Unethical Professional Behavior Tendencies

Most researches have tended to link person-centered characteristics and teachers’ unethical professional behaviors while ignoring the environment relevant factors (Zhang et al., 2021). Isolated individuals have their own distinct personal characteristics, and individuals who are integrated into the group are influenced by the group, with all of their values and behaviors (Giddings, 2002). Student teachers’ study is professionally learning-oriented. The professional ethics they receive are not only in the knowledge of textbooks but also in the groups they are exposed to in their learning process. Undoubtedly, the teacher who educated them has a great impact on them, not only their professional ethical values but also their future professional behaviors (Arslan and Dinç, 2017). Studies found that when student teachers are exposed to unethical professional behaviors by college teachers during professional learning, it also has significant intergenerational effects on future teachers (Mwanza et al., 2017; Wang et al., 2017). Unethical professional behaviors the student teachers experienced will motivate them to protect individual professional ethical values and lead them to change their values, even though the values are not correct (Arslan and Dinç, 2017). Student teachers are in the early stages of career exploration. Compared with those teachers who have many years of work experience, their professional ethical values and professional behavior tendencies are more susceptible to the professional environment they are exposed to.

Therefore, this study proposed Hypothesis 3 that the more teachers’ unethical professional behaviors that student teachers have experienced, the more unethical professional behavior tendencies they may display for themselves. Barrett et al.’s (2012) stated that the 24-item teachers’ unethical professional behaviors questionnaire includes two parts: professional ethical values and perceived frequency of unethical professional behaviors. Therefore, Barrett’s measure was used as a calibration questionnaire for the scale developed in this study.

### Student Teachers’ Gender Differences in Unethical Professional Behavior Tendencies

Ethical behaviors depend mainly on cognitive and emotional processes (Fumagalli et al., 2010). Most of the students have developed distinct cognition and emotion of the profession when they make professional choices, and the stereotype of the teacher is formed in the education stage (Vieira et al., 2017). Professional identity, as special cognition and emotion of practitioners for the profession, has a great influence on professional behaviors (Richter et al., 2021). Due to social role expectations, women who choose to be teachers are more socially conscious in the development of professional identity, and men pay more attention to professional skills and power (Croson and Gneezy, 2009; Cech, 2015). Male and female student teachers have different concerns about the same profession, and there may be some gender differences in the unethical professional behavior tendencies of student teachers. Studies have shown that women are driven by emotion, empathy, and care for others when they solve moral dilemmas, and they have greater empathy than men (Fumagalli et al., 2010; Baez et al., 2017); women are more sensitive and act more responsible in terms of unethical professional behavior tendencies in the workplace (Akinola et al., 2018). Teachers are considered to be similar to nurses and doctors (Morris et al., 2012). For student teachers who interact with students, higher empathy may make women avoid unethical professional behavior tendencies when dealing with work problems. Therefore, this study proposed Hypothesis 4 that there are gender differences in unethical professional behavior tendencies of student teachers, and that female student teachers engage in fewer unethical professional behavior tendencies. Measurement invariance is the precondition for comparing unethical professional behavior tendencies between male and female student teachers (Yusof et al., 2017). Therefore, in this study, measurement invariance of student teachers’ unethical professional behavior tendencies was assessed across gender, to provide a better basis for studying future student teachers’ unethical professional behavior tendencies.

### Overview of the Present Study

This study sought to develop a student teachers’ unethical professional behavior tendencies scale and assess its validity and reliability. Based on the previous studies about teachers and preservice teachers’ professional ethics, we proposed Hypothesis 1 that the student teachers’ unethical professional behavior tendencies scale has four dimensions: insults and discrimination, perfunctory attitude and carelessness, unfairness, and using power for personal gain. In addition to analyze the structural validity of the scale, this study also examined the interpretive, cultural, and population validity of the scale based on the indicators of cultural understanding described by Washington and McLoyd (1982). Specifically, interpretive validity, which gives priority to the cognition of the participants, is assessed by individual professional ethical values. It is that individuals who are best to be able to describe personal values set the stage for behavior tendencies. Therefore, we proposed Hypothesis 2 that personal professional ethical values significantly influence unethical professional behavior tendencies. Personal ethical values are closely related to socio-culture in the context of collectivist culture, and individuals’ ethical values are influenced by ethical behaviors around them (Kathrine, 2015; Malmi et al., 2020). Therefore, cultural validity is represented by the use of the perceived frequency of unethical professional behaviors which is concerned with identifying the rules. We proposed Hypothesis 3 that unethical professional behavior tendencies correlate with individual perceptions of the present frequency of unethical professional behaviors. Sound research practice would examine whether the construct generalizations may be appropriately made from one population to another. Population validity is assessed by the use of gender differences in this research which are too often ignored in unethical professional behavior tendencies research (Zhao, 2009), and we proposed Hypothesis 4 that female student teachers engage in fewer unethical professional behavior tendencies than male student teachers.

## Materials and Methods

### Participants

In the first sample, the initial unethical professional behavior tendencies scale included 17 items. One thousand one-hundred student teachers were investigated, and 979 questionnaires were collected (sample A). Among these, 287 were men (29.3%), with a mean age of 19.80 (SD = 1.34) and 692 were women (70.7%), with a mean age of 19.40 (SD = 1.17); 525 (53.6%) were freshmen, 293 (29.9%) were sophomores, and 161 (16.5%) were juniors. The whole sample was aged from 17 to 24 (*M* = 19.52, SD = 1.24), 141 male participants from the countryside, 145 male participants from the city, 347 female participants from the countryside, and 343 female participants from the city. Most of the participants belong to the Han ethnicity.

In the second sample, 1,165 student teachers were investigated. The revised student teachers’ unethical professional behavior tendencies scale included 12 items, the Barrett teachers’ unethical professional behavior questionnaire was also used, and 936 questionnaires were collected (sample B). Among them, 291 (31.1%) were men, with a mean age of 19.88 (SD = 1.30) and 645 (68.9%) were women, with a mean age of 19.49 (SD = 1.22); 504 (53.8%) were freshmen, 275 (29.4%) were sophomores, and 157 (16.8%) were juniors. The whole sample aged from 17 to 26 (*M* = 19.62, SD = 1.20), 150 male participants from countryside, 141 male participants from city. About 321 female participants from the countryside, 324 female participants from the city. Most of the participants belong to the Han ethnicity.

### Measures

The student teachers’ unethical professional behavior tendencies scale was revised based on the initial scale included 17 items developed by Wang et al. (2019), and each item started with “in some cases” to reduce participants’ guessing about the measurement purpose. All items are scored on a 5-point scale as *absolutely will not do* (1), *will not do* (2), *I do not know what to do* (3), *will do* (4), *absolutely will do* (5). Because student teachers lack practical teaching experience, the scale instruction reads, “If you are a teacher, how will you deal with the following problems? Please answer the following questions according to your actual situation.”

The 24-item teachers’ unethical professional behaviors questionnaire developed by Barrett et al. (2012) was used. The questionnaire includes four factors: personal harm, grade inflation, carelessness, and public/private boundary violations. For each item, respondents rated the extent to which they believed the behavior (a) occurred frequently and (b) represented a serious violation of professional standards. College teachers are the student teachers always in contact with, and they are the major group of student teachers perceived frequency of unethical professional behaviors in this study. All items are scored on a 5-point scale from 1 (absolutely disagree) to 5 (absolutely agree). It has been proved to possess good internal consistency reliability with all factors’ Cronbach’s *α* was higher than 0.76 in Barrett et al.’s (2012) research. The scale has been proved to possess good internal consistency reliability with Cronbach’s α of 0.962 (perceived frequency of unethical professional behaviors) and 0.978 (professional ethical values) for China student teachers (Wang, 2020). It also has been shown to possess good internal consistency reliability with all factors’ Cronbach’s α higher than 0.793 and good composite reliability with all factors’ McDonald’s *ω* higher than 0.807 in this study.

### Procedure

A lot of survey work was done before compiling this scale. (1) Thirty student teachers whose major is Chinese Language and Literature and Mathematics and English were interviewed to illustrate the deeply impressive teachers’ unethical professional behaviors they had experienced in primary and secondary school (Chinese, Mathematics, and English are the main subjects in primary and secondary school in China). (2) Thirty in-service teachers who teach student teachers were interviewed to illustrate the unethical professional behaviors that student teachers did most often (the 30 student teachers and 30 in-service teachers only participated in the interview survey). (3) This study reviewed the developed teachers’ unethical professional behaviors scales from China and other countries. (4) This study refers to the teachers” professional ethics regulations formulated by the Ministry of Education of the People’s Republic of China. Finally, the initial scale consists of 17 items (Table 1).

The research was carried out in accordance with the recommendations of the ethics committee of the School of Psychology, Jiangxi Normal University. The protocol was approved by the ethics committee of the School of Psychology, Jiangxi Normal University. Participants were student teachers without teaching practice experience, and they are learning how to be primary and secondary school teachers in the future. The investigation was carried out in accordance with the latest version of the Declaration of Helsinki. Participants were from a university in Jiangxi (the university is the largest university in the province to train student teachers), and all of them provided informed consent before the investigation. They completed the scale online during class time and were informed that they could drop out at any time. The survey was anonymous, and participants were told to fill out the scale according to their actual situation, and their true answers are very important to our study. Finally, they spent an average of 15 mins to complete the scale.

### Data Analytic Plan

Sample A (*N* = 979) was used for exploratory factor analysis (EFA) and item analysis. Sample B (*N* = 936) was used for confirmatory factor analysis (CFA) and assessment of concurrent validity. The total sample (*N* = 1,915) was used for the other analyses.

Exploratory factor analysis was conducted with the oblique rotation method. Three criteria were used as follows: (1) eigenvalue greater than 1, (2) no double/cross-loading, and (3) loading of 0.5 or higher on one factor. CFA was conducted to confirm the model from EFA. The following criteria were used to evaluate the overall fit. A value of 0.90 or higher for Tucker–Lewis index (TLI), comparative fit index (CFI), a root mean square error of approximation (RMSEA) of 0.08 or lower, and a standardized root mean square residual (SRMR) of 0.05 or lower served as estimates of adequate fit (Wang, 2014). McDonald’s *ω* was used to determine composite reliability, with 0.6 which represents the lowest acceptable value (McDonald, 1970; Hayes and Coutts, 2020). Cronbach’s *α* was used to determine internal consistency reliability, with 0.6 which represents the lowest acceptable value (Nunnally, 1978). Two-group CFA was conducted to examine the measurement invariance across gender (Wang, 2014). We checked each item to confirm whether they performed differently in subgroups (i.e., men vs. women, Clark and Watson, 2019).

Finally, we also examined the Pearson correlations between the unethical professional behavior tendencies and external variables (i.e., professional ethical values, individual perceptions of present frequency of unethical professional behaviors) using standards of weak (0.2–0.40), moderate (0.40–0.60), and strong (≥0.60; Dai et al., 2011).

The data were analyzed using the Statistical Program for SPSS Version 20.0, AMOS Version 17.0 and Jamovi Version 2.0.

## Results

### Item Analysis

Sample A (*N* = 979) was used for item analysis. Initially, participants’ scores on the 17 unethical professional behavior tendencies were divided into high and low groups by the total scores of the highest 27% and lowest 27%. The *t*-test results showed significant differences between the two groups in all 17 items (*p* < 0.001), and all 17 items had a *T* value greater than 3, reaching a very significant level. The correlation coefficient between each item and the total score ranged from 0.433 to 0.718 (*p* < 0.001). Therefore, all items showed good discrimination and were suitable for EFA.

### Exploratory Factor Analysis

Exploratory factor analysis was used with principal component analysis for sample A. The Kaiser–Meyer–Olkin (KMO) measure of sampling adequacy and Bartlett’s test of sphericity were examined to confirm the appropriateness of an EFA. The results indicated that the sample and correlation matrix were appropriate for factor analysis, with KMO index = 0.903, and Bartlett’s test of sphericity was statistically significant, *χ*^2^ = 3,846.754 (*N* = 979), *p* < 0.001.

In EFA, the following criteria were used: (a) eigenvalues greater than 1, (b) no double/cross-loadings, (c) loading of 0.5 or higher on one factor, and (d) conceptual clarity and theoretical salience. Five items were dropped as follows: the 4th, 5th, 8th, 10th, and 16th (refer to Table 1). EFA conducted using the data from the 12-item unethical professional behavior tendencies scale suggested that four factors should be extracted, which cumulatively accounted for 68.242% of the total variance. Each item loading ranged from 0.667 to 0.867. The four factors were named according to the item content: perfunctory attitude and carelessness (student teachers do not take education seriously during teaching), insults and discrimination (verbal humiliation or corporal punishment of students during study or life), unfairness (unfairly reward or punish students in their studies), and using power for personal gain (selling goods to the students or to be a tutor for gaining personal benefit, refer to Table 2).

**TABLE 2:** The 12-item unethical professional behavior tendencies scale EFA results.

**Item**	**Insults and discrimination**	**Perfunctory attitude and carelessness**	**Unfairness**	**Using power for personal gain**	**Factor loadings**
7. In some cases, humiliating students who disobey school rules	0.867				0.567
6. In some cases, ridiculing students	0.755				0.780
9. In some cases, discriminating against students based on family conditions	0.714				0.751
2. In some cases, treat students’ homework carelessly		0.857			0.651
3. In some cases, teach courses carelessly		0.833			0.798
1. In some cases, treat students carelessly		0.721			0.595
12. In some cases, reward students unfairly			0.818		0.566
13. In some cases, punish students unfairly			0.769		0.781
11. In some cases, use violent disciplinary methods with students who disobey school rules			0.678		0.761
15. In some cases, use teachers’ power for personal gain				0.786	0.614
14. In some cases, run tutorial groups for money				0.756	0.722
17. In some cases, selling school goods to students and their parents for money				0.667	0.602
Eigenvalue	2.208	2.166	1.992	1.824	
Cumulative contribution rate	18.397%	36.445%	53.045%	68.242%	

### Confirmatory Factor Analysis

Confirmatory factor analysis was conducted to confirm the bi-factor model for the 12 items, using sample B (*N* = 936), male data of sample B (*N* = 291), and female data of sample B (*N* = 645). We tested five models (refer to Table 3), and the maximum likelihood method was used to estimate these parameters. Results did not confirm Model A, Model B, and Model D. Model C, which included four correlated factors, fit the data adequately. However, the fit indices of Model E were better than those of Model C. Therefore, Model E, that is, the bi-factor 12-item unethical professional behavior tendencies scale, was the best fitting model (*χ*^2^/*df* = 3.775, SRMR = 0.044, RMSEA = 0.054, TLI = 0.956, CFI = 0.972, and AIC = 25,143.964).

**TABLE 3:** Fit indices for the five models tested.

**CFA model**	**χ^2^/*df***	**SRMR**	**TLI**	**CFI**	**RMSEA**	**90% CI**	**AIC**
Model A: one-factor (Total)	30.930	0.088	0.684	0.741	0.179	(0.171, 0.186)	26,557.241
Model A (male)	8.578	0.071	0.744	0.815	0.164	(0.148, 0.175)	8,806.446
Model A (female)	23.645	0.099	0.609	0.680	0.187	(0.179, 0.195)	17,455.319
Model B: uncorrelated four-factor (Total)	8.419	0.173	0.882	0.912	0.089	(0.081, 0.097)	25,509.608
Model B (male)	4.101	0.196	0.878	0.910	0.103	(0.089, 0.118)	8,618.510
Model B (female)	5.414	0.159	0.875	0.907	0.083	(0.073, 0.093)	16,607.055
Model C: correlated four-factor (Total)	5.493	0.043	0.953	0.965	0.069	(0.061, 0.078)	25,162.429
Model C (male)	2.959	0.046	0.942	0.957	0.082	(0.067, 0.098)	8,497.221
Model C (female)	3.988	0.044	0.948	0.962	0.068	(0.058, 0.078)	16,381.877
Model D: second-order (Total)	9.778	0.094	0.681	0.758	0.137	(0.126, 0.148)	13,889.701
Model D (male)	3.703	0.085	0.785	0.837	0.137	(0.116, 0.158)	4,571.960
Model D (female)	7.059	0.102	0.600	0.697	0.137	(0.124, 0.151)	8,976.805
Model E: bi-factor (Total)	3.775	0.044	0.956	0.972	0.054	(0.046,0.064)	25,143.964
Model E (male)	1.884	0.036	0.965	0.978	0.055	(0.036, 0.074)	8,473.828
Model E (female)	2.740	0.048	0.951	0.969	0.052	(0.041, 0.063)	33,084.848

*CFA, confirmatory factor analysis; SRMR, standardized root mean square residual; TLI, Tucker–Lewis index; CFI, comparative fit index; RMSEA, root mean square error of approximation; CI, confidence interval; and AIC, Akaike information criterion.*

### Testing for Gender Invariance

According to the results of EFA and CFA, we tested the gender invariance of the 12-items scale, using the total data (sample A and sample B). Results showed adequate fit for the bi-factor model of student teachers’ unethical professional behavior tendencies in both female (*N* = 1,337; *χ*^2^/*df* = 3.281; SRMR = 0.038; RMSEA = 0.041; TLI = 0.977; and CFI = 0.964) and male groups (*N* = 578; *χ*^2^/*df* = 2.572; SRMR = 0.052; RMSEA = 0.034; TLI = 0.973; and CFI = 0.957). The test of measurement invariance across gender results showed appropriate fit, as shown in Table 4. Using invariance testing, which is often used in the literature (Kern et al., 2018), constraints imposed did not worsen the model fit across the models. The full configural invariance model (Model 1) and metric invariance model (Model 2) supported measurement invariance (ΔCFI ≤ 0.010, ΔTLI ≤ 0.010), the full metric invariance model (Model 2) and full scalar invariance model (Model 3) supported measurement invariance (ΔCFI ≤ 0.010, ΔTLI ≤ 0.010), and the full scalar invariance model (Model 3) supported measurement invariance (ΔCFI ≤ 0.010, ΔTLI ≤ 0.010; Wang, 2014; Yusof et al., 2017). Although the full error variance invariance model (Model 4) did not support measurement invariance (ΔCFI ≤ 0.010, ΔTLI ≤ 0.010), invariance of the residuals is considered inconsequential in many types of research (Wang, 2014; Alatli, 2020). This research is interested in the comparison of observed mean differences between men and women and has no specific hypotheses about item uniqueness or reliability, and then demonstrations of metric and scalar invariance are critical and sufficient (Alatli, 2020). The final results indicated gender invariance of form, factor loadings, and item intercepts.

**TABLE 4:** Testing for gender invariance.

	**S-Bχ^2^**	***df***	**CFI**	**TLI**	**RMSEA (90% CI)**	**SRMR**	**ΔCFI**	**ΔTLI**
Model 1	345.474	96	0.963	0.949	0.052(0.046, 0.058)	0.044	–	–
Model 2	369.659	104	0.961	0.950	0.052(0.046, 0.057)	0.048	−0.002	0.001
Model 3	408.808	112	0.956	0.948	0.053(0.047, 0.058)	0.049	−0.005	−0.002
Model 4	695.928	124	0.915	0.910	0.069(0.064, 0.074)	0.057	−0.041	−0.038

*SRMR, standardized root mean square residual; TLI, Tucker–Lewis index; CFI, comparative fit index; and RMSEA, root mean square error of approximation.*

### Gender Differences in Student Teachers’ Unethical Professional Behavior Tendencies

Male student teachers had significantly higher average scores than female student teachers on all four factors and total unethical professional behavior tendencies, *p* < 0.05 (refer to Table 5). However, for the detection, the gender difference was not significant for perfunctory attitude and carelessness, and insults and discrimination, *p* > 0.05. For unfairness, male and female student teachers’ detection rates were 4.2 and 1.2%, respectively, and the difference was significant (*χ*^2^ = 17.236, *p* < 0.001). For using power for personal gain, male and female student teachers’ detection rates were 4.3 and 1.8%, respectively, and the difference was significant (*χ*^2^ = 10.362, *p* < 0.01).

**TABLE 5:** Gender differences in student teachers’ unethical professional behavior tendencies.

	**Male (*n* = 578)**	**Female (*n* = 1,337)**	**Mean difference**	***t***	**Cohen’s *d***
Total unethical professional behavior tendencies	23.692 ± 8.681	21.127 ± 6.663	2.565	6.341^***^	0.331
Perfunctory attitude and carelessness	6.952 ± 3.005	6.652 ± 2.717	0.299	2.058^*^	0.105
Insults and discrimination	4.483 ± 2.282	3.700 ± 1.471	0.783	7.592^***^	0.408
Unfairness	5.554 ± 2.805	4.546 ± 2.105	1.008	7.744^***^	0.406
Using power for personal gain	6.704 ± 2.720	6.229 ± 2.362	0.475	3.648^***^	0.186

*p < 0.05, and ***p < 0.001.

### Reliability and Construct Validity

We next tested the scale’s composite reliability and internal consistency reliability. Results showed that McDonald’s *ω* for the total scale (*ω* = 0.885) and the subscales (perfunctory attitude and carelessness = 0.833, insults and discrimination = 0.832, unfairness = 0.814, and using power for personal gain = 0.744) was satisfactory. Cronbach’s *α* for the total scale (*α* = 0.875) and the subscales (perfunctory and carelessness = 0.826, insults and discrimination = 0.813, unfairness = 0.806, and using power for personal gain = 0.705) was satisfactory (refer to McDonald’s ω and Cronbach’s *α* for men and women in Table 6). The results indicated that the scale had good composite reliability and internal consistency reliability.

**TABLE 6:** Composite reliability and consistency reliability of the unethical professional behavior tendencies scale.

**Dimensions**	**McDonald’s ω**			**Cronbach’s α**		
	**Total**	**Male**	**Female**	**Total**	**Male**	**Female**
Perfunctory attitude and carelessness	0.833	0.832	0.834	0.826	0.825	0.827
Insults and discrimination	0.832	0.828	0.825	0.813	0.812	0.803
Unfairness	0.814	0.829	0.792	0.806	0.822	0.781
Using power for personal gain	0.744	0.755	0.736	0.705	0.717	0.696
Total scale	0.885	0.896	0.872	0.875	0.890	0.859

The construct validity was assessed by examining the correlations between the total and the subscales. The correlations between the four factors were moderately significant (*r* = 0.396–0.611, *p* < 0.001, refer to in Table 7), the correlations between each factor and the total were strongly significant (*r* = 0.683–0.846, *p* < 0.001), and the correlations between each factor and the total both for male and female student teachers were strongly significant (*r* = 0.672–0.867, *p* < 0.001). The item-total correlations ranged from 0.525 to 0.746 (*p* < 0.001) and the mean inter-item correlations ranged from 0.126 to 0.699 (*p* < 0.01). The results showed that the four factors were independent and encompassed by overall unethical professional behaviors, which confirms the bi-factor model.

**TABLE 7:** The correlations between the total and the subscale.

	**Perfunctory attitude and carelessness**			**Insults and discrimination**			**Unfairness**			**Using power for personal gain**		
	**Total**	**Male**	**Female**	**Total**	**Male**	**Female**	**Total**	**Male**	**Female**	**Total**	**Male**	**Female**
Perfunctory attitude and carelessness	1	1	1									
Insults and discrimination	0.321^***^	0.399^***^	0.268^***^	1	1	1						
Unfairness	0.446^***^	0.496^***^	0.417^***^	0.642^***^	0.663^***^	0.609^***^	1	1	1			
Using power for personal gain	0.430^***^	0.458^***^	0.412^***^	0.512^***^	0.516^***^	0.505^***^	0.582^***^	0.612^***^	0.562^***^	1	1	1
Total of unethical professional behavior tendencies	0.683^***^	0.710^***^	0.672^***^	0.811^***^	0.833^***^	0.787^***^	0.846^***^	0.867^***^	0.825^***^	0.798^***^	0.793^***^	0.803^***^

***p < 0.001.

### Interpretive and Cultural Validity

The correlations between perceived frequency of unethical professional behaviors, professional ethical values, and the unethical professional behavior tendencies scale we developed were examined to assess concurrent validity. We found that the perceived frequency of unethical professional behaviors was significantly positively correlated with unethical professional behavior tendencies, whereas professional ethical values were significantly negatively correlated with unethical professional behavior tendencies (refer to Table 8). The correlations indicated that the more student teachers have experienced teachers’ unethical professional behaviors, the more unethical professional behavior tendencies they engage in themselves and that the better the student teachers’ professional ethical values, the less likely they are to engage in unethical professional behavior tendencies.

**TABLE 8:** Correlations between student teachers’ unethical professional behavior tendencies, professional ethical values, and perceived frequency of unethical professional behaviors (analysis based on a total sample).

		**Total of unethical professional behavior tendencies**	**Perfunctory attitude and carelessness**	**Insults and discrimination**	**Unfairness**	**Using power for personal gain**
Perceived frequency of unethical professional behaviors	Total of frequency	0.380^***^	0.273^***^	0.300^***^	0.323^***^	0.344^***^
	Personal harm	0.341^***^	0.229^***^	0.330^***^	0.300^***^	0.271^***^
	Carelessness	0.341^***^	0.241^***^	0.280^***^	0.295^***^	0.300^***^
	Public/private boundary violations	0.326^***^	0.243^***^	0.219^***^	0.271^***^	0.319^***^
	Grade inflation	0.337^***^	0.252^***^	0.239^***^	0.277^***^	0.321^***^
Student teachers’ professional ethical values	Total of ethical values	−0.170^***^	−0.173^***^	−0.094^**^	−0.109^**^	−0.161^***^
	Personal harm	−0.138^***^	−0.131^***^	−0.083^*^	−0.094^**^	−0.129^***^
	Carelessness	−0.154^***^	−0.168^***^	−0.094^**^	−0.092^**^	−0.131^***^
	Public/private boundary violations	−0.181^***^	−0.193^***^	−0.069^*^	−0.115^***^	−0.185^***^
	Grade inflation	−0.170^***^	−0.159^***^	−0.115^***^	−0.110^**^	−0.158^***^

*p < 0.05, **p < 0.01, and ***p < 0.001.

The correlations between female student teachers’ unethical professional behavior tendencies and their professional ethical values do not all fit hypotheses (refer to Table 9). Specifically, there has been no significant negative correlation between the insults and discrimination factor and professional ethical values.

**TABLE 9:** Correlations between student teachers’ unethical professional behavior tendencies, professional ethical values, and perceived frequency of unethical professional behaviors (Analysis based on male and female samples).

		**Total of unethical professional behavior tendencies**		**Perfunctory attitude and carelessness**		**Insults and discrimination**		**Unfairness**		**Using power for personal gain**	
**Gender**		**Male**	**Female**	**Male**	**Female**	**Male**	**Female**	**Male**	**Female**	**Male**	**Female**
Perceived frequency of unethical professional behaviors (M/F)	Total of frequency	0.441^***^	0.320^***^	0.320^***^	0.244^***^	0.407^***^	0.187^***^	0.348^***^	0.279^***^	0.428^***^	0.283^***^
	Personal harm	0.414^***^	0.271^***^	0.306^***^	0.184^***^	0.439^***^	0.217^***^	0.330^***^	0.251^***^	0.342^***^	0.215^***^
	Carelessness	0.372^***^	0.299^***^	0.278^***^	0.217^***^	0.348^***^	0.195^***^	0.273^***^	0.278^***^	0.368^***^	0.247^***^
	Public/private boundary violations	0.408^***^	0.256^***^	0.296^***^	0.210^***^	0.315^***^	0.116^**^	0.346^***^	0.199^**^	0.425^***^	0.250^***^
	Grade inflation	0.378^***^	0.302^***^	0.257^***^	0.246^***^	0.360^***^	0.137^***^	0.287^***^	0.258^***^	0.385^***^	0.280^***^
Professional ethical values (M/F)	Total of ethical values	−0.266^***^	−0.122^**^	−0.265^***^	−0.133^**^	−0.198^**^	–0.03	−0.213^***^	–0.054	−0.217^***^	−0.134^***^
	Personal harm	−0.260^***^	–0.071	−0.235^***^	−0.085^*^	−0.217^***^	0.006	−0.221^***^	–0.024	−0.205^***^	−0.092^*^
	Carelessness	−0.242^***^	−0.106^**^	−0.231^***^	−0.139^***^	−0.203^**^	–0.021	−0.185^**^	–0.038	−0.196^**^	−0.099^*^
	Public/private boundary violations	−0.241^***^	−0.144^***^	−0.270^***^	−0.156^***^	–0.112	–0.03	−0.193^**^	–0.066	−0.225^***^	−0.162^***^
	Grade inflation	−0.234^***^	−0.149^***^	−0.236^***^	−0.127^**^	−0.203^***^	–0.077	−0.183^**^	−0.081^**^	−0.165^**^	−0.160^***^

*p < 0.05, **p < 0.01, and ***p < 0.001.

## Discussion

This study revised a bi-factor model of student teachers’ unethical professional behavior tendencies that included four special factors (perfunctory attitude and carelessness, insults and discrimination, unfairness, and using power for personal gain) and one general factor (unethical professional behaviors), based on the previous studies (Ge, 2008; Barrett et al., 2012; Eren and Rakıcıoğlu-Söylemez, 2017; Zhang, 2017; Cai, 2019). The item analysis results showed the good discrimination of the scale and it had good internal consistency reliability for the total scale. Measurement invariance across gender was confirmed. The correlations between the perceived frequency of unethical professional behaviors, professional ethical values, and unethical professional behavior tendencies were consistent with our hypotheses. All data analyses suggested good reliability and validity for the student teachers’ unethical professional behavior tendencies bi-factor model, and the scale can be used to assess unethical professional behavior tendencies in student teachers. Besides, the process of insuring external validity is approached through the use of cultural, interpretive, and population validity, and both person-centered characteristics and environment-relevant factors were discussed.

The study found that student teachers’ unethical professional behavior tendencies are negatively correlated with their professional ethical values and positively correlated with the perceived frequency of unethical professional behaviors. That is the more that student teachers are exposed to teachers’ unethical professional behavior tendencies, the worse the student teachers’ professional ethical values, and the more unethical professional behavior tendencies they engage in. Professional ethical values affect personal attitude to the profession, and better professional ethical values could reduce unethical professional behavior tendencies (Cai, 2019). Whereas student teachers are in the process of learning how to become qualified teachers, their professional ethical values are easily influenced by the surrounding people and environment (Ssenyonga et al., 2019; Liu, 2021). Cultural validity is concerned with the process to identify the rules which regulate and conduct those rules which define various practices and institutions (Washington and McLoyd, 1982). Student teachers’ perceived frequency of unethical professional behaviors is a process to identify professional ethical rules. In this process, the professional behaviors of the teachers they are exposed to also have an intergenerational impact on their professional behaviors, just as the behaviors of employees in a business would be deeply influenced by the corporate culture (Villeval, 2014; Wang et al., 2017). Although the study did not prove the causal link between the two factors, this is a reminder that educators must not neglect the development of professional ethical values while cultivating the professional skills of student teachers, and that teaching is essentially a moral activity (Liu, 2021). Starting with the ethical values of the entire teaching industry is necessary for reducing the unethical professional behavior tendencies of student teachers, and student teachers should improve themselves in the process of receiving training to reduce their unethical professional behavior tendencies.

The study also found that male student teachers’ total unethical professional behavior tendencies and four factors’ scores were higher than those of female student teachers, consistent with our hypothesis. Regarding perfunctory attitude and carelessness, studies have found that women are more responsible than men at work (Akinola et al., 2018). Due to social expectations and various other reasons, female student teachers have a higher professional identity than male student teachers (Zhu and Wang, 2017); as professional identity influences professional investment (Lee and Eissenstat, 2018), higher professional identity encourages female student teachers to take their work more seriously. Differences in insults and discrimination may be explained by gender differences in human nature. Men are more inclined to make risky decisions at work. When student teachers face problems at work, men are more inclined to regard the problem as a challenge, so they engage in more extreme professional behaviors (Charness and Gneezy, 2012; Frick, 2021). Therefore, in the face of the problem of students, male student teachers are more likely to take excessive measures to manage their classes. Regarding unfairness and using power for personal gain, differences may be related to men having more aspirations for power and material gain in career development (Fumagalli et al., 2010; Baez et al., 2017). Primary and secondary school teachers typically face high stress and low-return situations, which are also important factors that lead to the gender ratio imbalance of student teachers in recent years. It is essential to increase the salary for teachers to improve the retention rate of teachers and maintain a more equal gender ratio of student teachers (Sak, 2018; Zavelevsky and Lishchinsky, 2020). The detection analysis also found that male student teachers had significantly higher detection rates than female student teachers in two factors (unfairness and using power for personal gain) and also prove the importance of cultivating ethical values and salaries to the teacher.

Perfunctory attitude and carelessness is the highest score and the highest detection of student teachers’ unethical professional behavior tendencies. This result may be related to the deeply ingrained nature of perfunctory attitude to students in the teaching profession. In Barrett et al.’s (2012) study, carelessness and unprofessionalism were considered to be the most common and least serious of teachers’ unethical professional behavior tendencies. Despite this, the professionalism of teachers is crucial to students in teaching (Richter et al., 2021). It is necessary to cultivate values that encourage the most basic careful, serious, and responsible attitudes to improve the professionalism of student teachers.

### Strength and Limitations

The bi-factor model of the student teachers’ unethical professional behavior tendencies scale was developed to reduce the “teacher-source” harm to students in teaching and life outside school. Student teachers lack teaching experiences, and the opportunity to observe their communication with students directly is rare. This scale can be used to assess the unethical professional behavior tendencies of student teachers and could play an important role in student teachers’ ethical cultivation. A strength of the study was the content of the student teachers’ unethical professional behavior tendencies scale in this study was comprehensive.

Perfunctory attitude and carelessness are regarded as less serious and are usually neglected, but the study shows that it is an important and independent factor of student teachers’ unethical professional behavior tendencies. We should challenge erroneous ideas at the outset to prevent problems before they can arise. Our study sample was large, and data were collected two times to make the results more reliable.

Nonetheless, there are several limitations in our study: first, the use of self-reports cannot rule out potential reporting bias, such as social expectations. Research has found that teacher self-reports are little different from students’ assessment of them (Arslan and Dinç, 2017). Second, the ethical values of student teachers may change and develop in the process of learning, but this study did not explore the different characteristics of student teachers in different grades. Future research could focus on the different characteristics of student teachers’ unethical professional behavior tendencies in different grades to help to identify the special ways to cultivate their ethical values. The observation could be used with scale (e.g., classroom observation during the internship period), which could make the research content more comprehensive and the research results more reliable.

## Data Availability Statement

The raw data supporting the conclusions of this article will be made available by the authors, without undue reservation.

## Ethics Statement

The studies involving human participants were reviewed and approved by the Ethics Committee of School of Psychology, Jiangxi Normal University. The patients/participants provided their written informed consent to participate in this study.

## Author Contributions

JW and X-QW: research framework design, development of the student teachers’ UPBT scale, data collation and analysis, and writing and revising the manuscript. J-YL, C-RZ, M-FL, and B-JY: revising the manuscript. All the authors contributed to the article and approved the submitted version.

## Conflict of Interest

The authors declare that the research was conducted in the absence of any commercial or financial relationships that could be construed as a potential conflict of interest.

## Publisher’s Note

All claims expressed in this article are solely those of the authors and do not necessarily represent those of their affiliated organizations, or those of the publisher, the editors and the reviewers. Any product that may be evaluated in this article, or claim that may be made by its manufacturer, is not guaranteed or endorsed by the publisher.
